# Acceleration of bone union by in situ-formed hydrogel containing bone morphogenetic protein-2 in a mouse refractory fracture model

**DOI:** 10.1186/s13018-020-01953-7

**Published:** 2020-09-18

**Authors:** Shintaro Shoji, Kentaro Uchida, Wataru Satio, Hiroyuki Sekiguchi, Gen Inoue, Masayuki Miyagi, Ken Takata, Yuji Yokozeki, Masashi Takaso

**Affiliations:** 1 Department of Orthopedic Surgery, Kitasato University School of Medicine, 1-15-1 Minami-ku, Kitasato, Sagamihara City, Kanagawa 252-0374 Japan; 2 Shonan University of Medical Sciences Research Institute, Nishikubo 500, Chigasaki City, Kanagawa 253-0083 Japan

**Keywords:** In situ-formed hydrogels, Hyaluronic acid, Bone morphogenetic protein, Refractory fracture

## Abstract

**Background:**

An enzymatic crosslinking strategy using hydrogen peroxide and horseradish peroxidase is receiving increasing attention for application with in situ-formed hydrogels (IFHs). Several studies have reported the application of IFHs in cell delivery and tissue engineering. IFHs may also be ideal carrier materials for bone repair, although their potential as a carrier for bone morphogenetic protein (BMP)-2 has yet to be examined. Here, we examined the effect of an IFH made of hyaluronic acid (IFH-HA) containing BMP-2 in promoting osteogenesis in a mouse refractory fracture model.

**Methods:**

Immediately following a fracture procedure, animals either received no treatment (control) or an injection of IFH-HA/PBS or IFH-HA containing 2 μg BMP-2 (IFH-HA/BMP-2) into the fracture site (*n* = 16, each treatment).

**Results:**

Fracture sites injected with IFH-HA/BMP-2 showed significantly greater bone volume, bone mineral content, and bone union compared with sites receiving no treatment or treated with IFH-HA/PBS alone (each *n* = 10). Gene expression levels of osteogenic markers, *Alpl*, *Bglap*, and *Osx*, were significantly raised in the IFH-HA/BMP-2 group compared to the IFH-HA/PBS and control groups (each *n* = 6).

**Conclusion:**

IFH-HA/BMP-2 may contribute to the treatment of refractory fractures.

## Background

Approximately 5 to 10% of fractures show poor healing because of delayed or non-union at the fracture site [1, 2]. When this arises, functional disabilities can result due to complications including pseudoarthrosis and skeletal deformities [3]. However, there are currently no effective treatments for refractory fractures. The use of bioactive materials that encourage bone formation and healing may improve intractable fracture healing.

One method used to increase the speed of fracture healing involves local application of growth factors [4–6]. For example, BMP-2, a potent osteoinductive cytokine, has the ability to induce bone and cartilage formation. However, because BMP-2 is only diffused via local administration, concerns have been raised regarding side effects such as attenuation of osteogenic potential and ectopic ossification [7, 8]. Therefore, growth factor delivery systems that sustain the release of BMP-2 at fracture sites are necessary to improve the success of bone healing and to limit possible side effects.

Implantable carriers such as absorbable collagen sponge or hydroxyapatite have been used to aid fracture healing in clinical settings. However, because these biomaterials require surgical incision for implantation, the method is invasive [9]. In contrast, injectable materials have the advantage of being less invasive than implantable materials. However, injectable materials generally diffuse only from the injection site compared to implantable materials [10]. Therefore, a material that is injectable and has the advantages of implantable materials may be an ideal candidate for a BMP-2 carrier.

An enzymatic crosslinking strategy using hydrogen peroxide (H_2_O_2_) and horseradish peroxidase (HRP) is receiving increasing attention for application with in situ-formed hydrogels (IFHs) made of natural polysaccharides, such as dextran, pullulan, and hyaluronic acid (HA) [11]. IFHs have suitable properties for biomedical application, including good cytocompatibility, tunable reaction rate, and substrate specificity [12–15]. Several studies have reported the application of IFHs in cell delivery and tissue engineering for bone or cartilage repair [12–16]. IFHs may also be ideal carrier materials for bone repair, although their potential as a carrier for BMP-2 has yet to be examined.

Glycosaminoglycans (GAGs) have been shown to improve BMP-2 bioactivity, including for obtaining the intended osteogenesis effects [17]. HA, a type of GAG, comprises linear polysaccharides made up of repeating disaccharide units of amino sugars and uronic acid, and is among the most used materials for enhancing the microenvironment for BMP-induced osteogenesis.

Here, we examined the effect of an IFH made of HA (IFH-HA) containing BMP-2 for promoting osteogenesis in a mouse refractory fracture model.

## Methods

### Preparation of hyaluronic acid (HA)-tyramine (TA) conjugate

The HA-TA conjugate was synthesized according to previously published methods [12, 18]. Briefly, TA (final concentration 12.5 mM; FUJIFILM Wako Pure Chemical Corporation, Osaka, Japan) was dissolved in 25 mM 50 kDa HA solution (Kewpie Corporation Fine Chemical Division, Tokyo, Japan), and 2.5 mM 1-ethyl-3-(3-dimethylaminopropyl) carbodiimide hydrochloride (FUJIFILM Wako Pure Chemical Corporation) and 2.5 mM N-hydroxysuccinimide (FUJIFILM Wako Pure Chemical Corporation) were added to start the conjugation reaction. Addition of 0.1 M NaOH was used to maintain the pH at 4.7 throughout the reaction. After stirring overnight at room temperature, the pH of the solution was raised to 7.0, and the solution was subsequently placed in dialysis tubes with a molecular cutoff of 1 kDa. Dialysis was performed in the tubes against 100 mM solution of sodium chloride for 2 days, a distilled water and ethanol mixture (3:1) for 1 day, and distilled water for 1 day. HA-TA was finally obtained by lyophilizing the purified solution.

### IFH-HA

IFH-HA was prepared by cross-linking HA-TA polymer in the presence of horseradish peroxidase (HRP; FUJIFILM Wako Pure Chemical Corporation), as the catalyzing enzyme, and H_2_O_2_ in 10 mM phosphate-buffered saline (PBS; pH 7.4). Briefly, HA-TA polymer solution (final concentration 2% w/v) was combined with 0.8 units/mL HRP solution (final concentration 0.8 units/mL) containing 2 μg BMP-2 (IFH-HA/BMP-2; PEPROTECH, Inc. Rocky Hill, NJ, USA) or PBS (IFH-HA/PBS) and H_2_O_2_ solution (final concentration 4 mM). Thus, the initial IFH-HA aqueous solution was cured through the HRP-H_2_O_2_ reaction (approximately 10 s).

### Sustained release of BMP-2 in vitro

To evaluate the sustained release of BMP-2 from IFH-HA, H_2_O_2_ solution containing HA-TA and HRP solution containing 2 μg BMP-2 were added to a 0.5-mL plastic microcentrifuge tube. After curing IFH-HA, 200 μl of PBS was added to the tube. To determine the release of BMP-2 from IFH-HA, BMP-2-loaded microtubes were incubated in 200 μl of PBS for 1, 4, 6, 24, 48, and 96 h, and 1, 2, and 3 weeks. The supernatant was collected and stored at − 30 °C until assay. The supernatant could not be collected after 6 weeks because the boundary between the gel and PBS was unclear. The concentration of BMP-2 in the supernatant was determined using the BMP-2 ELISA kit (R&D Systems, Inc., Minneapolis MN, USA). The experiment was repeated 5 times (*n* = 5).

### Mouse refractory fracture model

The femur fracture model was generated using 9-week-old C57BL/6 J mice [19]. The mice were maintained at Nippon Charles River Laboratories (Kanagawa, Japan) in a semi-barrier system under controlled temperature (23 ± 28 °C), humidity (55 ± 10%), and lighting (12-h light/dark cycle), and received standard rodent chow (CRF-1; Oriental Yeast, Tokyo, Japan). The refractory fracture model was generated by making a 10-mm incision in the lateral side of the left thigh under sterile conditions. The patella was medially dislocated by making a 4-mm lateral parapatellar incision. Following drilling of a 0.5-mm hole into the intercondylar notch, a stainless steel needle (0.5 mm in diameter) was retrogradely inserted into the intramedullary canal. Osteotomy was conducted using a wire saw (0.22 mm in diameter) via a small lateral approach, and insertion of a stainless steel needle into the intramedullary canal was used to stabilize the fracture. The entire circumference of an approximately 3-mm width of periosteum centered on the fracture site, except for the medullary space, was ablated using a bipolar electrocautery (AARON940, Bovie Medical Corporation, NY, USA) at a power setting of 1.2 W for 10 s to obtain an intractable fracture. Our preliminary study showed that a non-cauterized model achieved union at 4 weeks. In contrast, in the refractory fracture model, poor bone formation was observed in the cauterized region, and nonunion continued to be observed until 6 weeks after facture (Additional File 1). Therefore, this model was used as the refractory model in this study. Immediately following the fracture, 25 μl of IFH-HA/PBS or IFH-HA containing 2 μg BMP-2 (IFH-HA/BMP-2) was injected around the fracture using a microsyringe to ensure exposure of the entire fracture site and was subsequently stabilized around the injected site with curing (each *n* = 16). Animals that did not receive any treatment were used as controls (*n* = 16). Femurs from each of 10 mice in the control, IFH-HA/PBS, and IFH-HA/BMP-2 groups were used for micro-CT and histological analysis. The remaining 6 femurs in each group were analyzed by real time PCR analysis. All experimental procedures were performed in a blinded manner, with investigators blinded to the group assignment of each mouse. All animal experiments were conducted based on the guidelines of the Animal Ethics Committee of Kitasato University (approval number 2018-084).

### Determination of new bone volume and bone mineral content

All mice were sacrificed 6 weeks after treatment. Femurs along with the surrounding muscle were removed and fixed in 4% paraformaldehyde at 4 °C for 48 h. The femurs were moved into PBS and imaged on a micro-focus X-ray CT system (inspeXio SMX-90CT; Shimadzu, Tokyo, Japan) using a 90 kV acceleration voltage, 110 mA current, 20 lm/pixel voxel size, and 1024 × 1024 matrix size. Using the micro-CT images of whole femur, new bone volume and bone mineral content were quantified in a 3-mm region (cauterized region) of interest centered on the fracture site (150 slices) chosen at the mid femur for each animal using a three-dimensional (3D) image analysis software (Tri-3D-Bon; Ratoc System Engineering, Tokyo, Japan), as previously described. Regions of new bone were determined using a threshold density of 300 mg/cm^3^ [10, 19].

### Histology

After micro-CT analysis, the femurs were submerged in a carboxymethyl cellulose (CMC) gel and then placed in hexane, and subsequently frozen using solid CO_2_. The frozen blocks were attached to a CM 3050S IV cryomicrotome (Leica Instruments, Heidelberger, Germany) and cut using a disposable tungsten carbide blade in a cryochamber maintained at − 25 °C. The cut surface was covered with a Cryofilm (Finetec, Tokyo, Japan), and any air bubbles forming behind the film were removed using a brush. The blocks were cut into sections of 6-μm thickness using the tungsten carbide blade and dried at – 25 °C. Serial sections were stained with Von Kossa stain (Kureha Special Laboratory Co., Ltd., Tokyo, Japan). The area of new bone formed in a 3-mm region (cauterized region) of interest centered on the fracture site was quantified using the freehand tracing tool in ImageJ version 1.52 (National Institutes of Health, Bethesda, MD) (*n* = 10).

### Real time PCR

Six weeks after the fracture, we harvested calluses from the fracture site from the three groups (*n* = 6). Total RNA was extracted from calluses, and first-strand cDNA synthesis using SuperScript III RT (Invitrogen) was performed as described previously [20]. Gene expression of osteogenic markers, alkaline phosphatase (*Alpl*), osteocalcin (*Bglap*), and osterix (*Osx*), in callus was examined using real-time PCR. The following primers, designed using the Primer Blast software and synthesized by Hokkaido System Science Co. (Sapporo, Japan), were used for quantitative PCR: *Alpl*-sense 5′-CCACTATGTCTGGAACCGCA-3′ and *Alpl*-antisense 5′-GAGAGCGAAGGGTCAGTCAG-3′ (product size 132 bp); *Bglap*-sense 5′-AGTGTGAGCTTAACCCTGCT-3′ and *Bglap*-antisense 5′-ATAGATGCGTTTGTAGGCGGT-3′ (product size 74 bp); *Osx*-sense 5′-GCTGCGGGTATCCTGACTCT-3′ and *Osx-*antisense 5′-CGGTGGTAGTTACGGTCGG-3′ (product size 187 bp); *Gapdh*-sense 5′-AACTTTGGCATTGTGGAAGG-3′, and *Gapdh*-antisense 5′-ACACATTGGGGGTAGGAACA-3′ (product size 223 bp). Levels of messenger (m)RNA for each gene of interest were normalized against levels of *Gapdh* using the delta-delta Ct method.

### Statistical analysis

Differences among the control, IFH-HA/PBS, and IFH-HA/BMP-2 groups were examined using one way ANOVA with post hoc analysis (Tukey’s multiple comparison’s test). A *P* value of < 0.05 was considered statistically significant. Statistical analysis was performed using the SPPSS software (Version 25.0; SPSS, IBM, Armonk, NY, USA).

## Results

### Sustained release of BMP-2 from IFH-HA in vitro

**Fig. 1:**
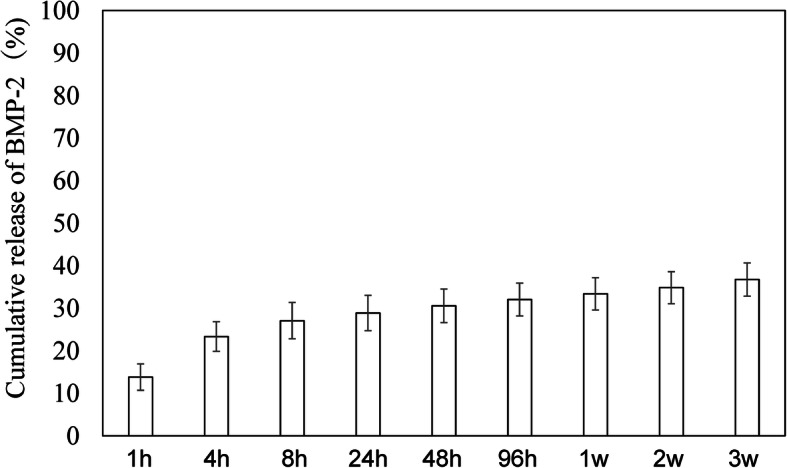
Sustained release of BMP-2 from IFH-HA in vitro. Cumulative release of BMP-2 in PBS at different time points. Results are presented as mean ± standard error (SE) (*n* = 5)

The in vitro release profile of BMP-2 from IFH-HA is shown in Fig. 1. BMP-2 release from HA gel occurred with an initial burst in the first 4 h followed by a gentler release pattern after 8 h. Thereafter, the sustained release rate was moderate, with 37% of the administered dose of BMP-2 gradually released across 3 weeks.

### HA gel containing BMP-2 induced callus formation in vivo

**Fig. 2:**
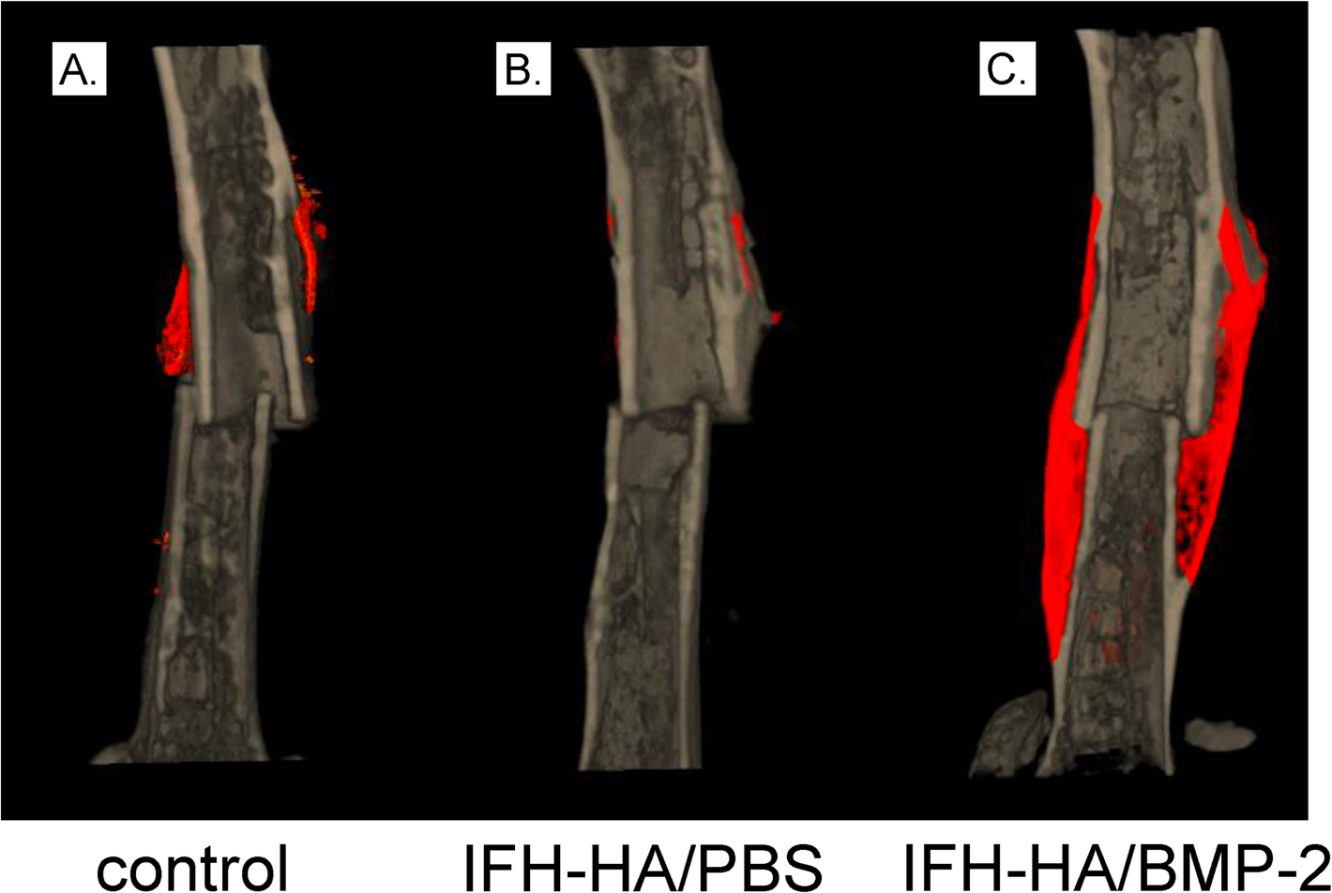
Representative 3D micro-CT image of femurs after injection of in situ-formed hyaluronic acid hydrogel (IFH-HA) loaded with BMP-2. 3D micro-CT images of fractured femurs from **a** control, **b** IFH-HA/PBS-, and **c** IFH-HA/BMP-2-treated groups after 6 weeks of recovery. Red, newly formed bone; gray, existing bone. Scale bars indicate 3 mm

**Fig. 3:**
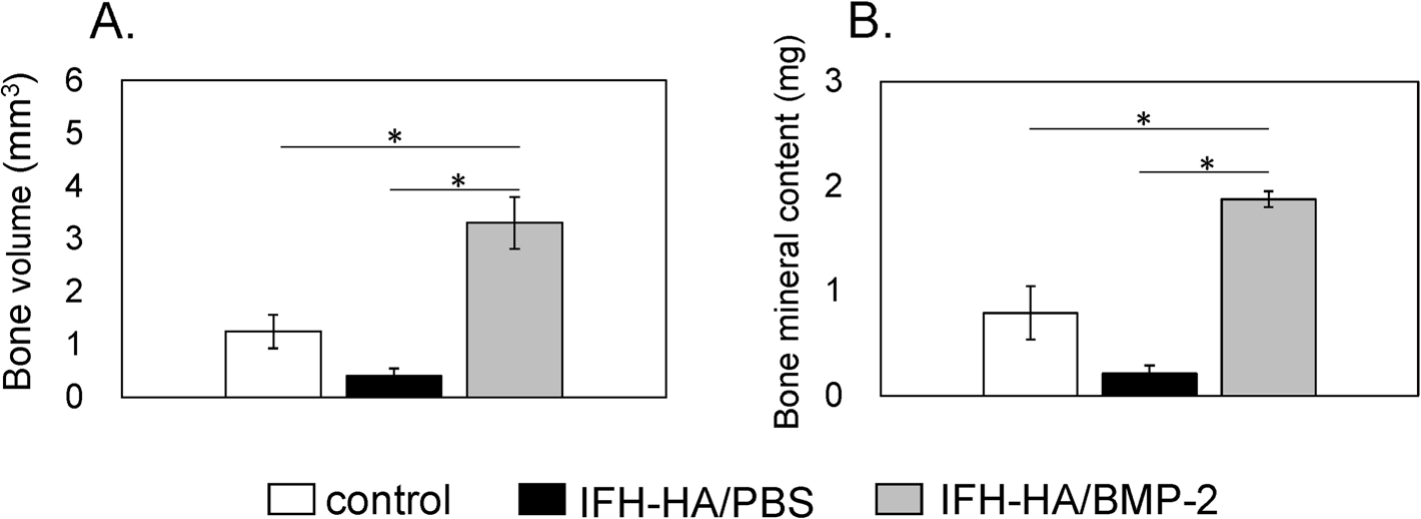
Quantification of the callus area and bone mineral content at the cauterized region in the fracture site 6 weeks after creation of the fracture. Analysis of **a** bone volume (mm^3^) and **b** bone mineral content (mg) in calluses from control (white bars), IFH-HA/PBS- (black bars), and IFH-HA/BMP-2-treated (gray bars) groups. Data are presented as the mean ± SE (*n* = 10). *p < 0.05 compared with the control group

We evaluated callus formation in fractured femurs following treatment with IFH-HA containing BMP-2 using micro-CT image analysis 6 weeks post-treatment (Figs. 2 and 3). Compared to sites that received no treatment (control) or treated with IFH-HA alone, fracture sites injected with IFH-HA/BMP-2 showed significantly greater bone volume and bone mineral content (*P* < 0.05). In contrast, bone volume and bone mineral content were comparable between the IFH-HA and control groups.

### Histomorphometric findings

**Fig. 4:**
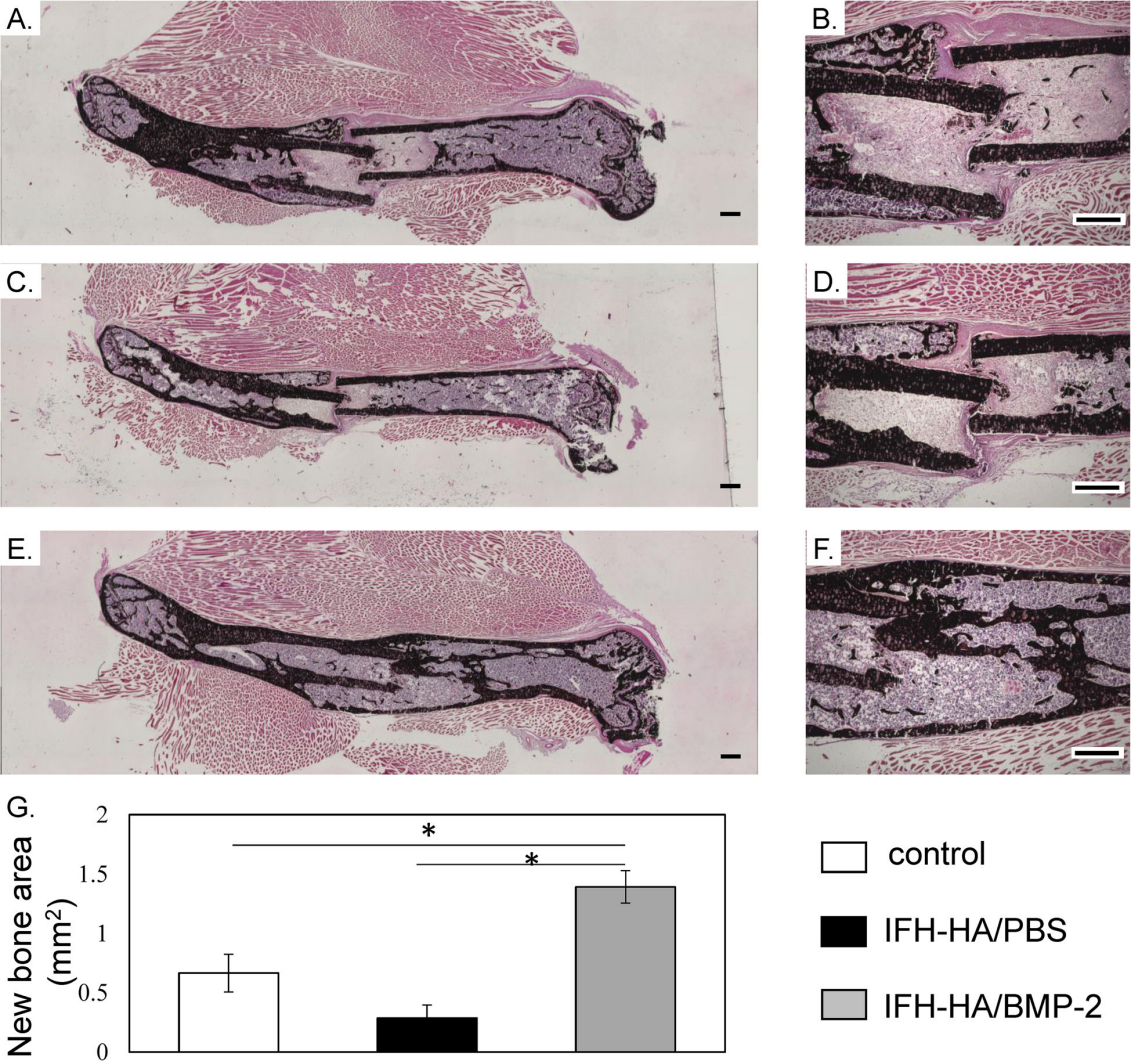
Von Kossa staining of undecalcified fresh-frozen sections of femur and surrounding muscle. Von Kossa staining of **a**, **b** control; **c**, **d** IFH-HA/PBS; and **e**, **f** IFH-HA/BMP-2. **g** Quantification of new bone at fracture sites. *p < 0.05. Scale bars indicate 500 μm. *p < 0.05

To evaluate bone union, we conducted histological examination of the fracture site 6 weeks post-fracture (Fig. 4a–f). The IFH-HA/BMP-2-treated group exhibited large calluses at the fracture site compared to the IFH-HA and control groups (*P* < 0.05, Fig. 4g), and the fracture site was bridged by newly formed bone (Fig. 4f). In contrast, in the IFH-HA and control groups, fibrous or cartilage callus tissue were observed at the fracture site, and there was no evidence of bone union (Fig. 4d, e).

### Expression of *Alpl*, *Bglap*, and *Osx*

**Fig. 5:**
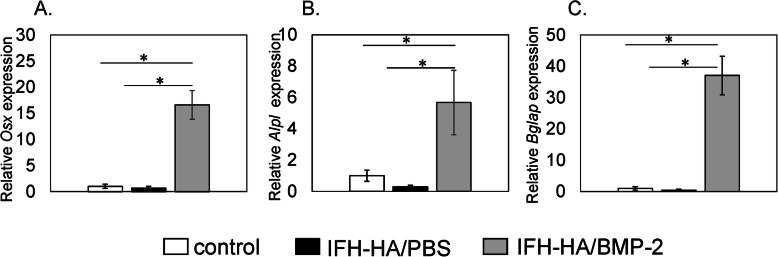
Gene expression of osteogenic markers in calluses. Real-time PCR analysis of the expression of *Osx* (**a**), *Alpl* (**b**), and *Bglap* (**c**) in calluses from control (white bars), IFH-HA/PBS- (black bars), and IFH-HA/BMP-2-treated (gray bars) groups (*n* = 6)

Gene expression levels of osteogenic makers, *Alpl*, *Bglap*, and *Osx*, were significantly raised in the IFH-HA/BMP-2 group compared to the IFH-HA and control groups (Fig. 5a, b, c; *P* < 0.05). Meanwhile, *Alpl*, *Bglap*, and *Osx* levels were comparable between the IFH-HA and control groups (*P* < 0.05).

## Discussion

Several studies have examined HA hydrogels for their potential as carriers for BMP-2. Injectable or implantable HA hydrogels retain BMP-2 and stimulate bone formation in vivo [17, 21–27]. Implantation of acrylated HA hydrogel with BMP-2 accelerates bone repair in calvarial defects in rats, and injectable hydrazone-crosslinked HA hydrogels with BMP-2 stimulate bone formation in rat calvaria [28]. In this study, we developed an injectable and immediately cured IFH/HA material. IFH-HA combined with BMP-2 promoted bone formation and bone union in a mouse refractory fracture model. IFH-HA, which possesses the advantages of injectable and implantable materials, may improve the success of bone healing and minimize the occurrence of possible side effects due to diffusion from HA hydrogel in situ. IFH-HA/BMP-2 may therefore be a promising material for promoting refractory fracture repair in clinical settings.

BMP-2 signaling is initiated early on in the first phase of bone healing and leads to inflammatory response and periosteal activation. BMP-2 is additionally important at later phases of chondro- and osteogenesis [29–31]. Therefore, long-term sustained release of BMP-2 is important for accelerating bone formation during fracture healing. In our study, 23% of the administered dose of BMP-2 was released from IFH-HA in 4 h. Subsequently, however, the release rate dropped to a moderate level, with approximately 100 ng/ml BMP-2—equivalent to the amount required to increase the ALP activity of mesenchymal stem cells [32, 33]—released between 2 to 3 weeks in vitro. BMP-2 stimulates osteoblastic differentiation via the transcription factor, *Osx* [33, 34]. In our study, mRNA expression of *Osx* and differentiation markers *Alpl* and *Bglap* were increased in calluses even 6 weeks after BMP-2 administration. Therefore, IFH-HA/BMP-2 may stimulate bone formation even at low doses in later phases of osteogenesis during fracture healing. Taken together, our findings suggest that IFH-HA/BMP-2 may be a useful material for enhancing refractory fracture healing in clinical settings.

Several limitations of the present study warrant mention. First, we did not determine the distribution of hydrogel or BMP-2 in vivo or the precise mechanism underlying the acceleration of bone formation following application of IFH-HA/BMP-2. Second, we did not conduct comparisons with previously reported bioactive materials [35–37]. Finally, future studies should test BMP-releasing hydrogel in critical size defects, where large quantities of new bone are needed and longer healing periods are available due to the nonunion nature of these defects.

## Conclusion

We examined the osteogenesis-promoting ability of HA gel containing BMP-2 in a mouse refractory fracture model. Fracture sites injected with HA/BMP-2 showed significantly greater bone volume, bone mineral content, and bone union compared with sites receiving no treatment or treated with HA alone. IFH-HA/BMP-2 may contribute to the treatment of refractory fractures.

## Supplementary information

**Additional file 1: Figure 1**. Fracture healing process in non-cauterized and cauterized fracture mouse models.

## Data Availability

The datasets supporting the conclusions of this article are included within the article. The raw data can be requested from the corresponding author.

## Abbreviations

BMP-2: Bone morphogenetic protein-2
HRP: Horseradish peroxidase
IFHs: In situ-formed hydrogels
HA: Hyaluronic acid
GAGs: Glycosaminoglycans
TA: Tyramine hydrochloride
CMC: Carboxymethyl cellulose
PCR: Polymerase chain reaction
Alpl: Alkaline phosphatase
Bglap: Osteocalcin
Osx: Osterix
ELISA: Enzyme-linked immunosorbent assay
SE: Standard error
