# Supplementary material for: Acceleration of bone union by in situ-formed hydrogel containing bone morphogenetic protein-2 in a mouse refractory fracture model
Source: J Orthop Surg Res. 2020 Sep 18;15:426. doi: 10.1186/s13018-020-01953-7 (PMC7501615; doi:10.1186/s13018-020-01953-7)
Supplement: Supplementary file 1 — Additional file 1: Figure 1. Fracture healing process in non-cauterized and cauterized fracture mouse models. [file 13018_2020_1953_MOESM1_ESM.docx]

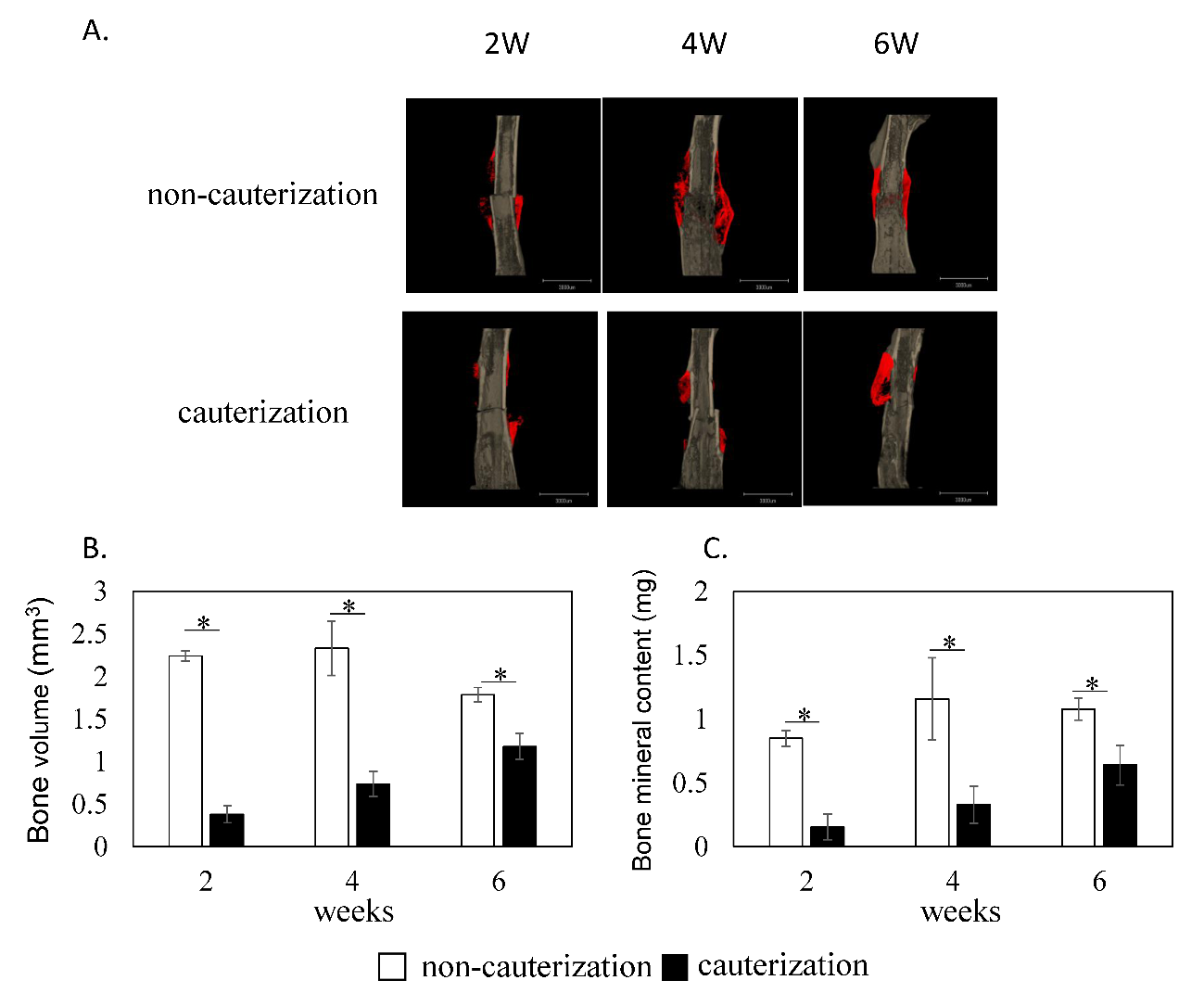


**Additional Figure 1. Fracture healing process in non-cauterized and cauterized fracture mouse models**

Micro-CT analysis of femurs from mice performed on a 3-mm region (cauterized region) of interest centered on the fracture site 2, 4 and 6 weeks after fracture in non-cauterized and cauterized fracture models (each n=5). (A) 3D micro-CT images of fractured femurs from non-cauterized and cauterized fracture models after 2, 4 and 6 weeks of recovery. Bone union in the non-cauterized group was achieved at 4 weeks after fracture. In contrast, non-union continued to be observed in the cauterized group even after 6 weeks post-fracture. (B–C) Analysis of bone volume (mm^3^) (B) and bone mineral content (mg) (C) in calluses from non-cauterized (white bars) and cauterized (black bars) groups. Bone volume and bone mineral content in the non-cauterized group were significantly higher than that in the cauterized group 2, 4 and 6 weeks after fracture. Data are presented as the mean ± SE (𝑛 = 5). *𝑃 < 0.05 compared with the non-cauterized group.
